# A Rare Case of Acquired Thrombotic Thrombocytopenic Purpura Triggered by Acute Pancreatitis

**DOI:** 10.7759/cureus.8477

**Published:** 2020-06-06

**Authors:** Metlapalli Venkata Sravanthi, Sharmil Suma Kumaran, Nishant Sharma, Padmaja Bojanapally

**Affiliations:** 1 Internal Medicine, The Wright Center for Graduate Medical Education, Scranton, USA; 2 Oncology, Hematology & Oncology Associates of Northeastern Pennsylvania, Scranton, USA

**Keywords:** ttp, acute pancreatitis, thrombocytopenia, maha, adamts13

## Abstract

Acquired thrombotic thrombocytopenic purpura (TTP) is a rare thrombotic microangiopathy with varied etiology and manifestations. It is uncommon for acute pancreatitis to trigger TTP. A 59-year-old man hospitalized with acute pancreatitis developed fever, acute kidney injury, thrombocytopenia, and microangiopathic hemolytic anemia (MAHA) on his second day in the hospital. Based on clinical suspicion and a high PLASMIC score indicating a severe deficiency in ADAMTS13 (a disintegrin-like and metalloprotease with thrombospondin type 1 motif no. 13) activity, a presumptive diagnosis of TTP was made. He was treated with plasmapheresis with improvement in his hemoglobin and platelet count. Severely deficient ADAMTS13 activity causing accumulation of large von Willebrand factor (VWF) multimers and subsequent formation of platelet rich microthrombi are thought to be the mechanisms of development of TTP. Proinflammatory mediators released during the systemic inflammatory response seen in acute pancreatitis can promote VWF activity and inhibit ADAMTS13 activity. Diffuse endothelial injury as a result of the inhibition of vascular endothelial growth factor (VEGF)-mediated endothelial protection as well as production of excessive reactive oxygen species during an episode of acute pancreatitis also contributes to the pathogenesis of TTP. Thrombocytopenia and MAHA in a systemic inflammatory state should raise the suspicion for TTP. The PLASMIC score can further aid in the diagnosis and early initiation of plasmapheresis, which is key to the outcome.

## Introduction

Acquired thrombotic thrombocytopenic purpura (TTP) is a rare, fatal thrombotic microangiopathy with an estimated incidence of three cases per 1,000,000 adults per year [1]. It is caused by severely reduced activity of von-Willebrand factor (VWF)-cleaving protease ADAMTS13 (acronym for ‘a disintegrin-like and metalloprotease with thrombospondin type 1 motif no. 13’). The disease manifests as thrombocytopenia, hemolytic anemia, and organ failures. Its etiology is unclear, although it has been linked to various conditions such as sepsis, autoimmune disorders, malignancies, and pregnancy. We report a rather rare presentation of acquired TTP triggered by acute pancreatitis. While acute pancreatitis is a well-documented complication of TTP, its potential to trigger TTP is less commonly seen, with only a few reported cases in our literature review.

## Case presentation

**Figure 1:**
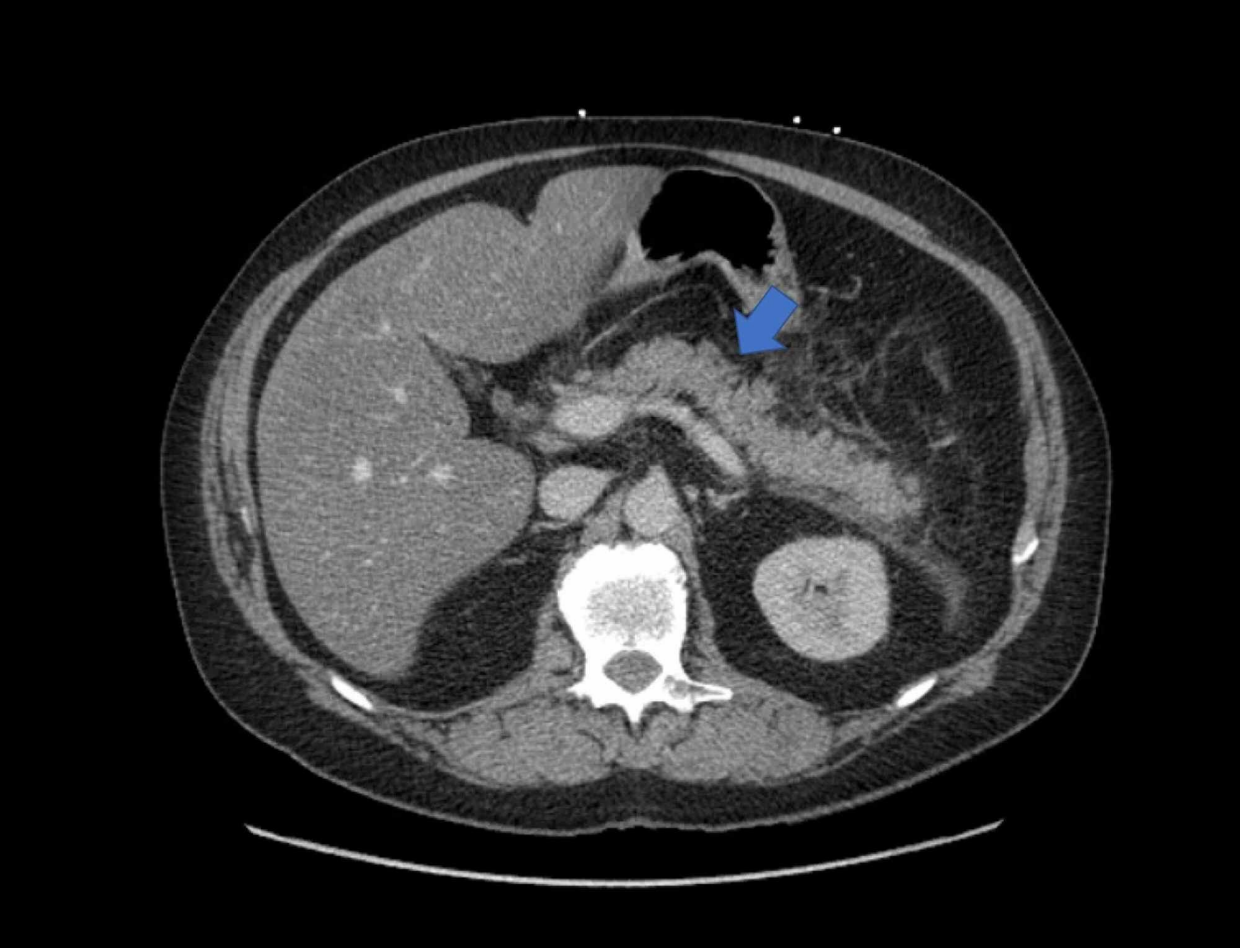
CT scan of abdomen demonstrating acute interstitial edematous pancreatitis.

A 59-year-old male with a history of type 2 diabetes, hypertension, dyslipidemia, bipolar disorder, and daily alcohol use, presented with acute onset, severe epigastric abdominal pain radiating to the back, associated with nausea and vomiting. Physical examination was notable only for epigastric tenderness. Pertinent labs include neutrophilic leukocytosis (13.2 x 109/L; reference range 4.5-10 x 109/L), elevated lipase (2353 units/L; reference range 0-160 units/L), and lactic acidosis (2.4 mmol/L; reference range. 0.4-2 mmol/L). CT scan of the abdomen was notable for interstitial pancreatic edema and inflammatory changes, suggestive of acute pancreatitis (Figure 1). He was treated conservatively with bowel rest, intravenous hydration, and opioid analgesics, with satisfactory symptomatic improvement. On hospital day two, he developed fever, stage II acute kidney injury, thrombocytopenia, and his hemoglobin dropped by 2.6 g/dL. Over the next four days, his hemoglobin and platelets down trended to nadirs of 6.8 g/dL (reference range 14.0-16.8 g/dL) and 42 x 109/L (reference range 150-400 x 109/L) respectively. An elevated lactate dehydrogenase (LDH) (1118 units/L; reference range 0-250 units/L) and low haptoglobin level (< 1 µmol/L; reference range 3-20 µmol/L) suggested a hemolytic process. A peripheral smear revealed schistocytes (2 per high power field) confirming the hemolysis. A negative direct antiglobulin test ruled out autoimmune hemolytic anemia. An elevated fibrinogen level and normal D-dimer level argued against disseminated intravascular coagulation. A negative serotonin release assay did not favor heparin-induced thrombocytopenia. Further imaging and cultures ruled out an infectious etiology. A PLASMIC score of 6 was calculated, indicating a high risk (72%) of severe ADAMTS13 deficiency (<15%).

A presumptive diagnosis of acquired TTP was made, and he was treated with four sessions of plasmapheresis, in addition to intravenous steroids and supportive blood transfusions. His platelet counts and renal function steadily improved with subsequent normalization. Rise in hemoglobin was trailing behind that of platelet count, but on a follow up a month later it was found to be closer to the normal range.

## Discussion

Von-Willebrand factor is a large glycoprotein produced as a homopolymer in endothelial cells, megakaryocytes, and subendothelial connective tissue. The primary function of VWF is to promote hemostasis. It binds to platelet GP1b-IX-V receptor complex and subendothelial collagen, resulting in platelet adhesion to the subendothelium as well as platelet GPIIb/IIIa receptors, allowing platelet-platelet aggregation. Shear stress in arteriolar and capillary circulation triggers VWF and platelets to form aggregates. VWF also acts as the carrier for factor VIII. The endothelial VWF polymer is cleaved into multimers of progressively smaller size by ADAMTS13, a plasma metalloprotease. The smaller multimers are hemostatically less effective than larger multimers [2].

Inflammatory conditions can cause decrease in ADAMTS13 activity; various mechanisms have been postulated, such as transcriptional down regulation, proteolytic degradation, and consumption due to the high plasma VWF levels in systemic inflammation [3]. Severely reduced ADAMTS13 activity results in abnormalities in VWF multimer quantities and patterns, leading to a prothrombotic state [4]. Endothelial injury and platelet aggregation have been implicated in the formation of platelet thrombi rich in VWF form in terminal arterioles and capillaries [2, 5]. The widespread platelet thrombi formation leads to thrombocytopenia. Red blood cells are subjected to shearing stress when they pass through the microcirculation burdened with thrombi, and consequently undergo intravascular hemolysis, resulting in anemia. These thrombi are extensively found in microcirculation of heart, brain, kidney, pancreas, spleen, mesentery and adrenal glands, resulting in varied clinical manifestations [2]. 

A systematic review of patient data from Oklahoma TTP registry by Page et al. found that all patients had thrombocytopenia and microangiopathic hemolytic anemia (MAHA), 80% had neurologic manifestations, 52% had renal abnormalities, and 10% had fever [6]. The classic ‘pentad’ of MAHA, thrombocytopenia, fever, acute renal failure, and neurologic abnormalities were rare, found only in less than 5% patients. Another systematic review by Hawkins et. al. reported cardiac involvement in TTP [7]. Gastrointestinal involvement in TTP is common [6]. An inciting event is not usually identified in the majority of the cases. While pancreatic involvement in TTP is an established entity, acute pancreatitis as an inciting event of TTP is sparsely reported, with only 11 case reports and two case series found in our literature review. Swisher et al., in a review of five such cases, found a median interval of three days between the diagnosis of acute pancreatitis and TTP, corresponding to the timeline in this patient [8]. Another review of such cases from a regional TTP registry in UK also found a median interval of three days [9]. The majority of the cases in the former series had a clear etiology, whereas most cases in the latter were idiopathic. In both case series, pancreatitis was resolving when the signs of TTP first appeared.

Acute pancreatitis can trigger a systemic inflammatory response with increased plasma levels of cytokines such as tumor necrosis factor‐α (TNF‐α), interleukin (IL)‐1, IL‐6, and IL‐8 [10-11]. These pro-inflammatory cytokines released by activated endothelium are implicated in endothelial injury, which in turn has been postulated to be a causative mechanism of TTP [8, 10-11]. IL‐6 and IL‐8 levels peak three days after the onset of pancreatitis, further implicating their role in development of TTP given the median interval between onset of pancreatitis and TTP is also three days. Studies in animal and human models have demonstrated an increased expression of vascular endothelial growth factor (VEGF) in the early phase of acute pancreatitis [12]. VEGF increases vascular permeability contributing to systemic inflammatory manifestations. Some animal experiments also suggested a protective role of VEGF against endothelial injury [13]. A decoy receptor of VEGF, soluble fms-like tyrosine kinase 1 (sFlt-1), has been strongly associated with the severity of pancreatitis [14]. Thus, a corresponding increase in its decoy receptor concentrations could prevent the endothelial protective effects of VEGF. Also, scavenging of reactive oxygen species produced by activated endothelium is hindered in acute pancreatitis, causing damage to endothelial cells [11]. The enzymes released by pancreas, leaking into circulation can also cause endothelial injury [11].

Angiopoietin-2 stored in Weibel-Palade bodies of endothelial cells is an indicator of endothelial injury and has been found to be in high concentrations in acute pancreatitis [10]. The major protein stored in Weibel-Palade bodies is vWF. IL-8 and TNF stimulated the release of the ultra large form of vWF from the endothelium in in vitro studies [15]. Additionally, IL-6 inhibits cleavage of ultra large VWF by ADAMTS13 under shear stress [15]. Several studies demonstrated elevated vWF in acute pancreatitis [11,16]. Morioka et al. reported increased concentrations of vWF with a mean of 402% on admission, which is in concordance with a report of low activity of ADAMTS13 among 13 severe acute pancreatitis patients without disseminated intravascular coagulation (DIC) [17]. ADAMTS13 activity has been known to be reduced in systemic inflammation, through mechanisms stated earlier. ADAMTS13 IgG titer was insignificant in all patients in the UK TTP registry case series; it is unlikely to be involved in pathogenesis of TTP in these cases [9]. Increased vWF concentrations positively correlated with severity of organ failure, APACHE III scores, and sequential organ failure assessment (SOFA) scores [16]. Conversely, decreased ADAMTS13 concentrations negatively correlated with APACHE II scores and pancreatic enzyme levels [17]. Development of TTP could be a consequence of the elevated VWF level and decreased ADAMTS13 activity caused by a systemic inflammatory state associated with pancreatitis. It is unlikely that pancreatitis was a clinical manifestation of TTP; in the majority of reported cases hemoglobin and platelets were normal or near normal on presentation and TTP occurred when pancreatitis was resolving. Due to the rarity of this association, it is difficult to delineate the exact chain of events; further research is needed on this association.

Thrombocytopenia and MAHA are early clues to TTP that can be tested for easily, quickly, and inexpensively. An elevated LDH, low haptoglobin, and presence of two or more schistocytes per high power field suggest MAHA. The sensitivity of the clinical pentad is too low to be of any help. The PLASMIC score is a useful tool to predict the likelihood of low ADAMTS13 activity in adults, and is an aid for the diagnosis of TTP. It takes into consideration factors such as platelet count, presence of hemolysis, mean corpuscular volume (MCV), international normalization ratio (INR), serum creatinine, presence of cancer, and presence of solid organ or stem cell transplants. A score of six or more strongly indicates severe deficiency of ADAMTS13 activity (less than 15%). This scoring system is highly sensitive and is superior to standard clinical judgement in predicting severe ADAMTS13 deficiency, assisting in the diagnostic challenge of TTP [18]. Severely deficient ADAMTS13 activity supports the diagnosis, but has a long turnaround time. Therapy is initiated based on clinical diagnosis. Plasma exchange (PEX) is the mainstay of therapy [19]. Glucocorticoids and rituximab improve outcomes and reduce the duration of plasma exchange [20]. Early initiation of plasmapheresis is key to the clinical outcome and survival rates are excellent in such cases. A high index of clinical suspicion should be maintained in a patient with hemolytic anemia and thrombocytopenia, despite the nature of presentation; timely diagnosis and treatment is paramount, given the high fatality rate.

## Conclusions

Acute pancreatitis is a well-documented consequence of TTP. However, acute pancreatitis leading to TTP is rarely reported. Systemic inflammation associated with acute pancreatitis appears to be involved in the pathogenesis of TTP in these cases. Thrombocytopenia and MAHA are the most consistent findings in the majority of reported cases. The classic description of TTP ‘pentad’ or ADMATS13 activity assay is not reliable in making a diagnosis sufficiently early in the disease process. Regardless of the nature of presentation, new onset thrombocytopenia and MAHA in a systemic inflammatory state, should forewarn of a potential diagnosis of TTP.
